# Spontaneous preterm delivery is reflected in both early neonatal and maternal gut microbiota

**DOI:** 10.1038/s41390-021-01663-8

**Published:** 2021-08-04

**Authors:** Henni Hiltunen, Maria Carmen Collado, Helena Ollila, Terhi Kolari, Satu Tölkkö, Erika Isolauri, Seppo Salminen, Samuli Rautava

**Affiliations:** 1 Department of Pediatrics, University of Turku, Turku, Finland; 2 Department of Pediatrics and Adolescent Medicine, Turku University Hospital, Turku, Finland; 3 Institute of Agrochemistry and Food Technology-National Research Council (IATA-CSIC), Valencia, Spain; 4 Functional Foods Forum, University of Turku, Turku, Finland; 5 Department of Public Health, University of Turku and Clinical Research Centre, Turku University Hospital, Turku, Finland; 6 Department of Biostatistics, Department of Clinical Medicine, University of Turku, Turku, Finland

## Abstract

**Background:**

Aberrant gut microbiota composition in preterm neonates is linked to adverse health consequences. Little is known about the impact of perinatal factors or maternal gut microbiota on initial preterm gut colonization.

**Methods:**

Fecal samples were collected from 55 preterm neonates (<35 gestational weeks), 51 mothers, and 25 full-term neonates during the first 3–4 postpartum days. Gut microbiota composition was assessed using 16S ribosomal RNA gene sequencing.

**Results:**

Preterm neonates exhibited significantly lower gut microbiota alpha diversity and distinct beta diversity clustering compared to term neonates. Spontaneous preterm birth was associated with distinct initial gut microbiota beta diversity as compared to iatrogenic delivery. Gestational age or delivery mode had no impact on the preterm gut microbiota composition. The cause of preterm delivery was also reflected in the maternal gut microbiota composition. The contribution of maternal gut microbiota to initial preterm gut colonization was more pronounced after spontaneous delivery than iatrogenic delivery and not dependent on delivery mode.

**Conclusions:**

The initial preterm gut microbiota is distinct from term microbiota. Spontaneous preterm birth is reflected in the early neonatal and maternal gut microbiota. Transmission of gut microbes from mother to neonate is determined by spontaneous preterm delivery, but not by mode of birth.

**Impact:**

- The initial gut microbiota in preterm neonates is distinct from those born full term. Spontaneous preterm birth is associated with changes in the gut microbiota composition of both preterm neonates and their mothers. The contribution of the maternal gut microbiota to initial neonatal gut colonization was more pronounced after spontaneous preterm delivery as compared to iatrogenic preterm delivery and not dependent on delivery mode.
- Our study provides new evidence regarding the early gut colonization patterns in preterm infants.
- Altered preterm gut microbiota has been linked to adverse health consequences and may provide a target for early intervention.

## Introduction

Preterm birth, a global challenge with significant health consequences,^1^ affects ~10% of pregnancies worldwide. While the exact cause for spontaneous preterm delivery can often not be identified, bacterial infection and conditions related to disturbances in the maternal microbiota such as inflammatory bowel disease, poor dental health, or bacterial vaginosis have been associated with preterm delivery risk.^2–4^ Recent studies suggest that more subtle alterations in the maternal gut microbiota composition may also be associated with an increased risk of spontaneous preterm birth.^5,6^ In addition to its potential role in spontaneous preterm delivery, the maternal gut microbiota is one of the most important factors shaping the initial gut colonization of the newborn.^7,8^ The compositional development of the vaginally delivered full-term and breast-fed child’s gut microbiota has been relatively well characterized, while in the preterm counterpart, our understanding of the microbiota establishment remains incomplete.

The preterm gut microbiota profile differs from full-term gut microbiota with decreased diversity and a higher abundance of proinflammatory bacteria.^9^ The immediate perinatal exposures in preterm neonates, including birth by cesarean section, frequent antibiotic exposure, lack of skin-to-skin contact, and breast milk, may disturb early gut colonization. Interestingly, initial gut colonization in preterm neonates appears to be distinct, as the microbiota composition of meconium, defined as the first stool passed and reflecting the life in utero, has been reported to be different in preterm as compared to term neonates.^10^ However, it is not known whether the microbiota alterations defined in the preterm neonate are associated with factors causing preterm birth, the preterm birth per se, immaturity, or whether they are mere consequences of the different treatment and feeding procedures.

Specific microbial features of the preterm gut microbiota have been linked to a higher risk for common preterm short-term morbidities including necrotizing enterocolitis (NEC)^11,12^ and poor postnatal growth.^13–15^ Understanding the early colonization process in the preterm gut may provide new means to prevent these complications. In the present study, we focused on the initial microbial pioneers in order to address the early differences in gut microbiota between preterm and full-term neonates and the factors influencing the gut microbiota establishment in preterm neonates. In particular, the purpose of this study was to investigate whether the cause of preterm birth and the maternal gut microbiota affect the microbial composition of preterm gut microbiota. In addition, the contribution of other factors potentially affecting the early preterm gut microbiota composition including gestational age, mode of delivery, intrapartum antibiotics, and intrauterine growth was investigated.

## Materials and methods

### Participants and sample collection

**Fig. 1:**
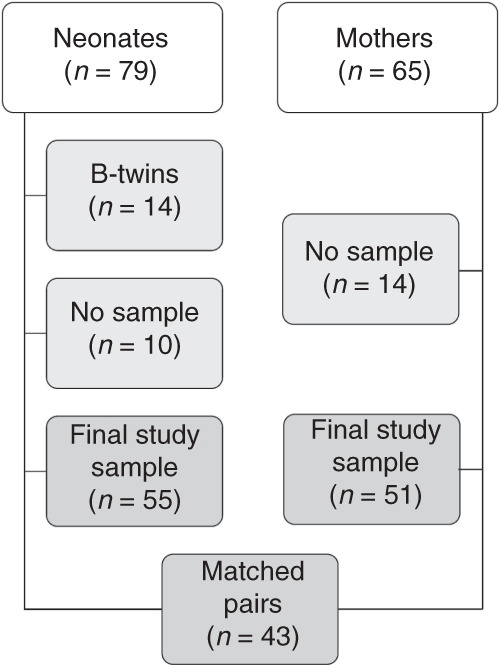
Flowchart of the study population exclusion process and matched newborn-mother pairs. Excluded study subjects are shown in light grey, and included study subjects in dark grey. In addition, the number of matched newborn-mother pairs is shown.

The study was conducted prospectively in the Turku University Hospital in Turku, Finland. For the preterm group, we recruited mothers (*n* = 65) and their newborns (*n* = 79) with a duration of gestation <35 weeks. The threshold of 35 gestational weeks was chosen to include a clearly preterm population, admitted in the neonatal intensive care unit (NICU) and receiving uniform treatment according to the hospital protocol. A substantial number of preterm neonates with a gestational age of 35–37 weeks are not admitted in the NICU, but treated in the maternity ward together with the mother, and therefore this population was excluded. Children with severe congenital anomalies were excluded. Also, the children (*n* = 10) and mothers (*n* = 14) without adequate samples as well as B-twins (*n* = 14) were excluded, resulting in a total of 55 preterm neonates and 51 mothers. The exclusion process as well as matched newborn–mother pairs are presented in Fig. 1. In addition, 25 fecal samples from spontaneously born term newborns were collected for comparison. The study was found ethically acceptable by the Ethics Committee of the Hospital District of Southwest Finland. Written informed consent was obtained from the caregivers.

To analyze the initial gut microbiota composition, preterm neonate stool samples were collected directly after birth, within the first 3 days of life. The maternal stool samples were collected within 3 days postpartum. The samples from term neonates were collected 3–4 days postpartum. Sample collection was conducted during the neonatal intensive care and maternity ward period at the Turku University Hospital. The samples were frozen and stored at −80 °C until analysis.

For the preterm population, the cause of preterm birth (spontaneous, preterm premature rupture of membranes (PPROM) or iatrogenic) was collected from patient records. In this study, iatrogenic preterm delivery was defined as delivery due to a maternal or fetal reason, i.e., pre-eclampsia, placental insufficiency, or hepatogestosis without spontaneous rupture on membranes or onset of labor. In addition, data regarding the following clinical characteristics were obtained: gestational age, sex, mode of delivery, intrapartum and neonatal antibiotic use, birth weight (grams and *Z*-score), whether they were small for gestational age (birth weight below the 10th percentile, SGA), starting day for enteral feeding (mother’s own or donor milk), and starting day of mother’s own milk. Similar data regarding the term neonates were collected.

### DNA extraction and 16S ribosomal RNA (rRNA) amplicon sequencing

Total DNA was extracted from the biological samples as previously described.^16^ Briefly, 100–125 mg of feces was weighed and homogenized in the presence of lysis buffer via bead beating with FastPrep-24 (MP Biomedicals, Irvine, CA). DNA was extracted following the commercial kit InviMag Stool DNA Kit (Stratec Molecular, Berlin, Germany) using the automated KingFisher DNA System (Thermo Fisher Scientific Oy, Vantaa, Finland). The KingFisher’s system protocol steps included nucleic acid binding on magnetic beads, five-step washing, and elution. Then, the total DNA concentration was measured using a Qubit® 2.0 Fluorometer (Life Technology, Carlsbad, CA) and normalized. A specific 16S rRNA gene region (V3–V4 region) was amplified following the 16S rDNA gene Metagenomic Sequencing Library Preparation Illumina protocol (Cod. 15044223 Rev. A). After 16S rDNA gene amplification, the multiplexing step was performed using Nextera XT Index Kit (FC-131-2001). One microliter of the PCR product was run on a Bioanalyzer DNA 1000 chip to verify the size; the expected size on a Bioanalyzer trace is ~550 bp. The libraries were sequenced using a 2 × 300 bp paired-end run (MiSeq Reagent Kit v3) on a MiSeq-Illumina platform according to the manufacturer’s instructions.

### Bioinformatics and statistical analysis

The raw sequences were analyzed with QIIME2 (version 2019-7 and 2020-11) pipeline. The data were imported using the Phred33-importing tool for paired-end data and quality filtered using the DADA2 method. The taxonomy was formed by using the Greengenes v.13.8 database and 99% amplicon sequence variant taxonomic classifier creating a phylogenetic tree. Alpha diversity was assessed using Faith’s Phylogenic Diversity; in addition, alpha-diversity evenness and Shannon indexes were used. Beta diversity was conducted using Bray–Curtis and Unweighted Unifrac distance matrices. Analysis of composition of microbes (ANCOM)^17^ was used to study differences in the taxonomical abundances between the groups. Group comparison was conducted with either analysis of variance or permutational multivariate analysis of variance statistical analysis. The statistical significance was determined as a corrected *p* value of <0.05.^18^ Calypso software version 8.24 (http://cgenome.net/calypso/) was used, along with total sum normalization for the statistical analysis.

The contribution of maternal gut microbiota composition on the preterm gut microbiota composition was selected as a response variable. Relationships between the percentage of microbes and continuous variables (gestational age, birth weight *Z*-score, maternal and neonatal alpha diversity as assessed by Shannon index) were examined with Spearman’s rank correlation coefficient because of non-normal distribution. Associations between the relative abundances of microbial taxa and categorical variables were analyzed with Wilcoxon’s rank-sum test. The cause of prematurity (iatrogenic/spontaneous), mode of delivery (vaginally delivered/cesarean section), intrapartum antibiotic exposure (yes/no), and neonatal antibiotic exposure (yes/no) were treated as categorical variables. The level of significance was set at *p* value <0.05. The analyses were performed with the SAS software, version 9.4 for Windows (SAS Institute Inc., Cary, NC).

## Results

### The clinical characteristics of the preterm and term neonates

The clinical characteristics of the preterm study subjects born after spontaneous or iatrogenic delivery are presented in Table 1. Vaginal birth was more common in the subjects born spontaneously (*p* < 0.001), whereas the neonates born by iatrogenic preterm delivery had lower birth weight (*p* = 0.046) and were more often small for gestational age (*p* = 0.002). All neonates in the study received human milk at the time of sample collection. None of the subjects had received the formula. There were three cases of chorioamnionitis in the preterm study population, with a premature delivery at 30, 31, and 34 weeks of gestation, and out of these cases, two newborns and one mother participated in the analyses.

**Table 1:** The data are presented as means (SD) for continuous variables and as numbers (percentage) for categorical variables.

	All	Spontaneous	Iatrogenic	*p* value
*N*	55	34	21	
Gestational age, weeks	32.28 (1.75)	32.28 (1.74)	32.29 (1.81)	0.99
Male	22 (40 %)	14 (41 %)	8 (38 %)	0.82
Vaginal delivery	34 (62 %)	28 (82 %)	6 (29 %)	<0.001
Intrapartum antibiotics	32 (59 %)	23 (70 %)	9 (43 %)	0.050
Neonatal antibiotics	51 (93 %)	30 (88 %)	21 (100 %)	0.29^b^
Birth weight, g	1885 (544)	1951 (526)	1780 (569)	0.26
Birth weight, *Z*-score	−0.27 (1.53)	0.064 (1.28)	−0.79 (1.76)	0.046
SGA 10th percentile	13 (24 %)	3 (9 %)	10 (48 %)	0.002^b^
Starting day for enteral nutrition^a^	1st (1st, 1st)	1st (1st, 1st)	1st (1st, 1st)	1
Starting day for mother’s own milk^a^	2nd (2nd, 3rd)	2nd (2nd, 3rd)	2nd (2nd, 3rd)	0.76
Days of parenteral nutrition^a^	4 (2, 6)	3 (1, 6)	4 (2, 6)	0.19

Differences between subjects born by spontaneous or iatrogenic preterm delivery were compared using Student’s *t* test for continuous variables and *χ*^2^ test for categorical variables. SGA = small for gestational age.

^a^Median (quartiles Q1, Q3) and Wilcoxon’s rank-sum test was used because of the exception of normal distribution.

^b^Fisher’s exact test was used because of the small cell numbers.

The characteristics of the term population are presented in Table 2. The term neonates were mainly vaginally delivered. All the term neonates received mother’s own milk from the first day of life. It is notable that none of them received antibiotics.

**Table 2:** Characteristics of the term population.

	All
*N*	25
Gestational age, weeks	40.83 (0.75)
Male	9 (36 %)
Vaginal delivery	23 (92 %)
Intrapartum antibiotics	5 (20 %)
Neonatal antibiotics	0 (0 %)
Birth weight, g	3479 (477)
Birth weight, *Z*-score	0.9 (1.27)

The data are presented as means (SD) for continuous variables and as numbers (percentage) for categorical variables.

### The initial preterm gut microbiota compared to the initial term gut microbiota

**Fig. 2:**
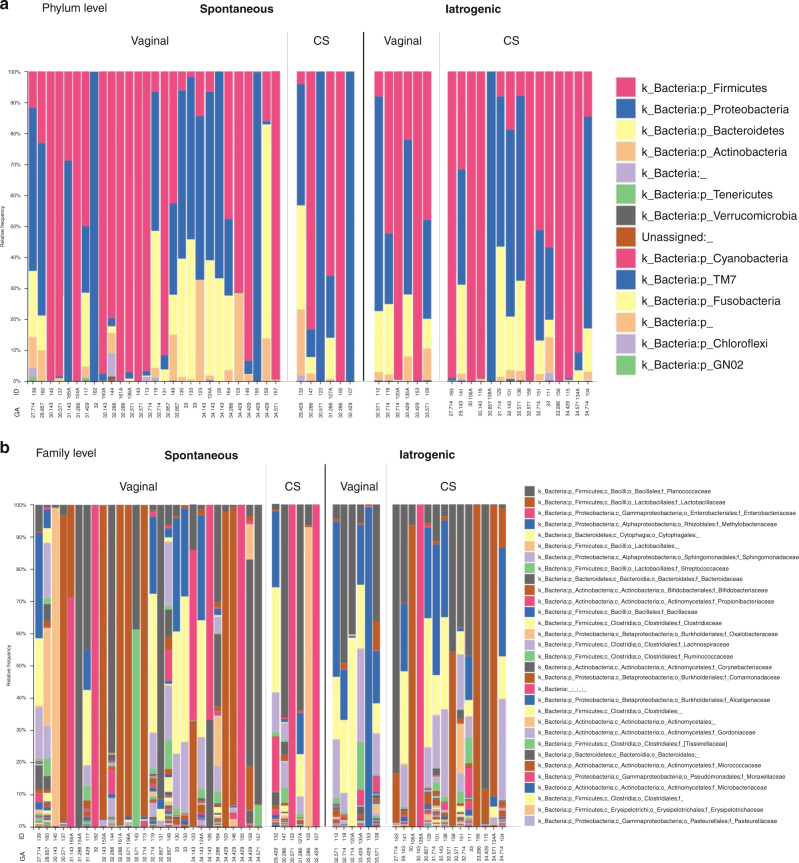
Preterm gut microbiota taxa plots. Preterm gut microbiota taxa plots at (**a**) phylum and (**b**) family levels are grouped by gestational age (GA), cause of prematurity and mode of delivery. The most abundant phyla were Firmicutes, Proteobacteria and Bacteroidetes, and the most abundant families were Planococcaceae, Lactobacillaceae and Enterobacteriaceae. The 31 most abundant families are included.

The preterm neonate (*n* = 55) initial gut microbiota composition was compared to the initial gut microbiota composition of spontaneously born, term neonates (*n* = 25). The preterm gut microbiota was mainly composed of bacteria belonging to the phyla Firmicutes (60.4%), Proteobacteria (26.3%), and Bacteroidetes (8.8%) (Fig. 2a). The three most abundant bacterial families in the preterm gut microbiota were Planococcaceae (28.2%), Lactobacillaceae (20.8%), and Enterobacteriaceae (11.7%) (Fig. 2b). Wide variation was seen among the preterm subjects in the taxonomic gut microbiota composition. In the term population, at the phylum level, the gut microbiota was mainly composed of Proteobacteria (32.5%), Firmicutes (30.7%), and Actinobacteria (29.5%) (Supplementary Figure 1A). The three most abundant bacterial families were Bifidobacteriaceae (30.6%), Enterobacteriaceae (24.9%), and Planococcaceae (15.4%) (Supplementary Figure 1B).

**Fig. 3:**
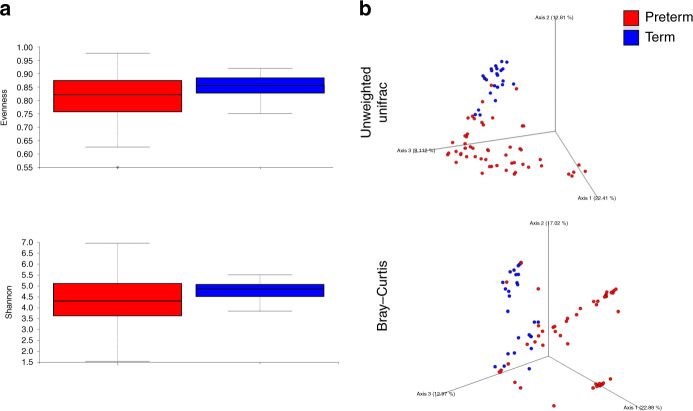
Comparison between the preterm and term gut microbiota composition. Significant differences were observed in **a** alpha-diversity evenness (*p* = 0.02) and Shannon (*p* = 0.03) (Kruskall–Wallis test) and **b** beta diversity as assessed by Unweighted Unifrac (*p* = 0.001) and Bray–Curtis (*p* = 0.001) (PERMANOVA) tests.

Significant differences were observed in both alpha and beta diversity as well as in specific relative abundancies when comparing the two groups. Significantly higher alpha diversity was observed in the term population compared to the preterm population as assessed by alpha-diversity evenness (*p* = 0.02) and Shannon index (*p* = 0.03) (Fig. 3a). Significant clustering was seen in Bray–Curtis (*p* = 0.001) and Unweighted Unifrac (*p* = 0.001) beta-diversity matrices according to the two groups (Fig. 3b). In the ANCOM analysis, at the phylum level, Actinobacteria were more abundant in the term group (W = 13). At the family level, the term gut microbiota exhibited higher levels of Bifidobacteriaceae (W = 104), Streptococcaceae (W = 103), and Bacteroidaceae (W = 102) compared to those observed in preterm. At the genus level, there were statistically higher levels of *Bifidobacterium* (W = 179), *Streptococcus* (W = 178), and *Bacteroidetes* (W = 177) in the term microbiota compared to preterm.

### The effect of perinatal factors and maternal gut microbiota on the initial preterm gut microbiota

**Fig. 4:**
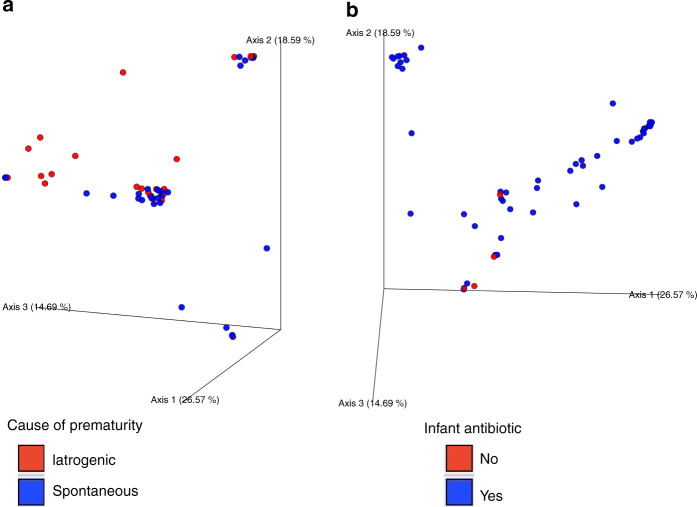
Preterm gut microbiota beta diversity PCoA plots. Significant clustering in PCoA plots of preterm neonate gut microbiota beta diversity grouped by (**a**) cause of prematurity (*p* = 0.047) and (**b**) neonatal antibiotic exposure (*p* = 0.045). The *p*-values correspond to the Bray Curtis test.

Significant clustering of the gut microbiota in the preterm neonates was observed according to the cause of prematurity (*p* = 0.047) (Fig. 4a) and postnatal exposure to antibiotics (*p* = 0.045) (Fig. 4b) as assessed by Bray–Curtis beta diversity. No differences in alpha diversity or Unweighted Unifrac beta diversity were seen in relation to these factors. Gestational age, mode of delivery, intrapartum antibiotic exposure, or intrauterine growth retardation did not affect the initial preterm gut microbiota composition as assessed by Faith Phylogenetic Diversity, alpha-diversity evenness and Shannon index alpha diversity (Supplementary Figure 2), or Bray–Curtis or Unweighted Unifrac beta diversity. As assessed with ANCOM, no consistent patterns or significant differences in the relative abundances of specific taxa were detected at phylum, family or genus levels in neonates born after spontaneous and iatrogenic preterm delivery, nor were gestational age, mode of delivery, intrapartum antibiotic exposure, or intrauterine growth retardation associated with relative taxonomic differences.

**Fig. 5:**
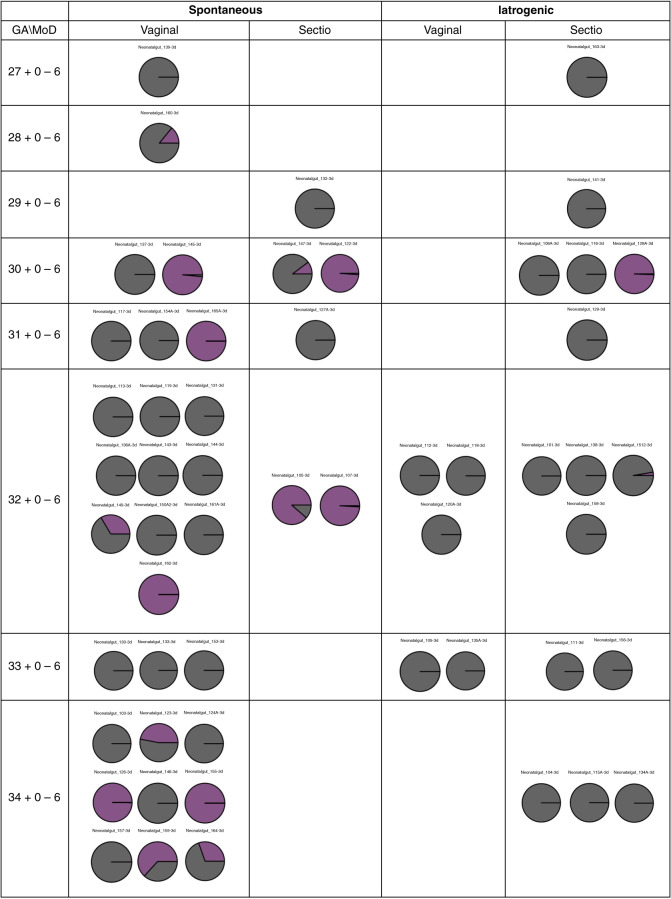
The contribution of the maternal gut microbiota to the initial neonatal gut microbiota composition as assessed by Source Tracker analysis and grouped by gestational age (GA), cause of prematurity, and mode of delivery (MoD). Each chart represents one study subject. The maternal contribution is calculated as a percentage and shown in purple.

The contribution of the maternal gut microbiota composition to the preterm gut microbiota composition was assessed with Source Tracker using QIIME version 1.9 (qiime.org/tutorials/source_tracking.html) (Fig. 5). The rate of the maternal gut microbiota contribution to the preterm gut microbiota varied markedly between individuals, but was higher in neonates born spontaneously as compared to those born after iatrogenic preterm delivery (*p* = 0.007). It is of note that gestational age or mode of delivery did not affect the extent to which the maternal gut microbiota contributed to the preterm gut microbiota. Indeed, a manifest contribution from the maternal gut microbiota was also seen in the microbiota of neonates born by cesarean section.

### The maternal gut microbiota in preterm birth

In the maternal gut microbiota, Firmicutes (62.4%) was the dominant taxa at the phylum level, followed by Bacteroidetes (29.6%) and Actinobacteria (4.3%) (Supplementary Figure 3A). At the family level, Bacteroidaceae (22.5%), Lachnospiraceae (22.4%), and Ruminococcaceae (21.6%) were most abundant (Supplementary Figure 3B). In the ANCOM analysis, the mothers who had received intrapartum antibiotics exhibited a higher abundance of Firmicutes (W = 2), Fusobacteria (W = 0), Proteobacteria (W = 1), and Actinobacteria (W = 0) as compared to nonexposed mothers. The mothers not receiving intrapartum antibiotic had a higher abundance of Porphyromonadaceae (W = 37). At the genus level, mothers with vaginal delivery presented a higher abundance of *Roseburia* (W = 118) and mothers without antibiotic treatment a higher abundance of *Macellibacteroides* (W = 63).

**Fig. 6:**
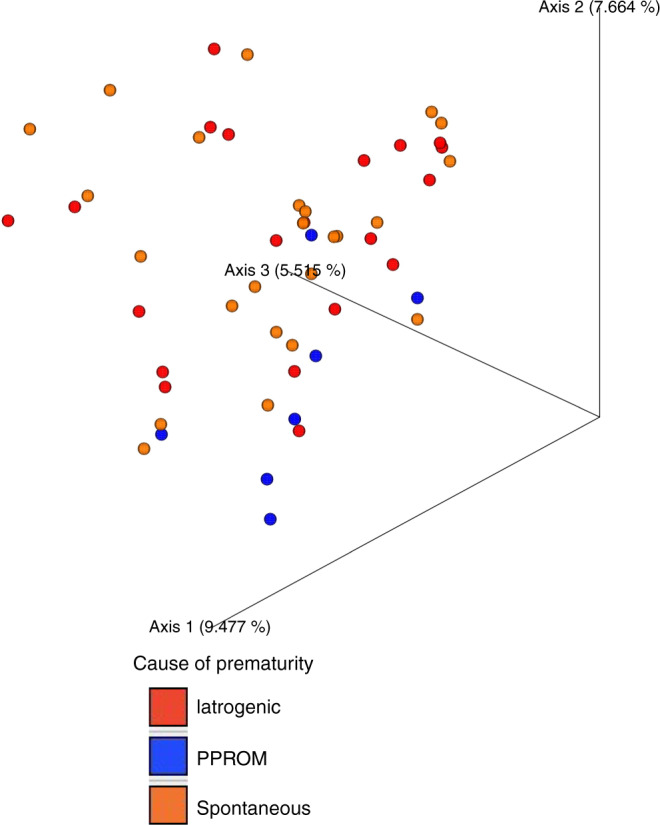
PCoA plots of maternal microbiota beta diversity grouped by cause of prematurity. There was a statistically significant difference between mothers with spontaneous delivery and PPROM delivery (*p* = 0.041) as assessed by the Bray–Curtis test.

Distinct and significant clustering of the gut microbiota was detected among mothers who delivered spontaneously, iatrogenically or after PPROM delivery by Bray–Curtis beta-diversity analysis (*p* = 0.041) (Fig. 6). There were no statistical differences in alpha or beta diversity in relation to gestational age or mode of delivery. The maternal gut microbiota composition was associated with intrapartum antibiotic use, which was to be expected in samples collected postpartum. Statistically significant differences with regard to intrapartum antibiotic use were seen in alpha-diversity Faith PD (*p* = 0.039) and evenness (*p* = 0.003), as well as Bray–Curtis (*p* = 0.004) and Unweighted Unifrac beta diversity (*p* = 0.002).

## Discussion

The initial gut microbiota in preterm neonates is distinct from that of those born full-term. The aberrant gut colonization in preterm neonates may at least in part be explained by detrimental perinatal and postnatal exposures, including cesarean section delivery, antibiotic use, and delayed or reduced exposure to breast milk, which often cluster in these fragile individuals. However, we have previously reported that prematurity per se affects the preterm gut microbiota composition.^19^ The present study for the first time provides data indicating that spontaneous preterm birth, often triggered by infection or other maternal microbial perturbation, may also affect the initial gut colonization in preterm neonates. Two distinct clusters were seen according to the cause of prematurity. However, there was nonsignificant variation in the ANCOM analysis in these two groups, not forming a consistent colonization pattern. Interestingly, other perinatal exposures such as intrapartum antibiotic use, delivery mode, or gestational age were not associated with the initial preterm gut microbiota composition in the present study. Furthermore, our results suggest that the initial preterm gut microbiota might at least in part originate from the time in utero as differences were seen in both preterm neonatal and maternal samples according to cause of prematurity. Several possible maternal–fetal transmission routes have been suggested.^20^ Changes in the gut microbiota in mothers with spontaneous preterm delivery may therefore affect early preterm gut colonization.

Significant differences in the gut microbiota were detected between mothers after spontaneous preterm delivery as compared to iatrogenic preterm delivery. Previously, numerous studies have indicated that changes in maternal microecology resulting from bacterial vaginosis, inflammatory bowel disease, or poor dental health may be associated with an increased risk for preterm birth.^2–4^ More recently, it has been shown that alterations in the maternal gut microbiota composition may also be associated with an increased risk of spontaneous preterm birth.^5,6^ Taken together, our study suggests that the events leading to spontaneous preterm birth are reflected not only in the gut microbiota of the mother but also of the newborn. This notion is corroborated by the higher contribution of the maternal gut microbiota to the neonatal gut microbiota after spontaneous preterm birth.

It has previously been extensively shown that the mode of delivery is a strong modulator of the early gut microbiota in term infants.^21^ In the present study, however, the preterm children born with cesarean section did not differ from vaginally delivered in terms of the gut microbiota composition. This observation is consistent with the studies by Dahl et al.,^22^ as well as Ardissone et al.,^10^ who have provided evidence suggesting that the impact of delivery mode is not substantial in the gut colonization process in preterm neonates. Notably, bacteria usually connected with the birth canal and vaginal birth (Lactobacillaceae family) were detected in the early gut microbiota of preterm neonates born both by vaginal delivery and by cesarean section in the present study. Furthermore, no differences in the contribution of the maternal gut microbiota to the preterm neonate gut microbiota were detected between subjects born by vaginal or cesarean section delivery in the Source Tracker analyses. This suggests that the early colonization of the preterm gut does not follow the same patterns as in term infants.

The relatively small sample size may affect the applicability of the results. However, the study subjects were characterized in detail, and a structured study protocol was followed while collecting the samples and treating the preterm neonates in the NICU. All the preterm neonates received breast milk, either from donors or their own mothers, from the first day of life as recommended by the European Society of Paediatric Gastroenterology, Hepatology and Nutrition. Antibiotics were used according to the unit protocol; initial empirical antibiotics consisted of a combination of penicillin G and gentamicin. The term population chosen for the comparison was a uniform group with no neonatal antibiotic treatment, and the term fecal samples were collected during the first days of life post partum. A limitation is that the preterm samples were collected 0 to 3 days postpartum, and therefore possibly promoting a higher microbial variation and a lower bacterial biomass. In addition, the maternal fecal samples were collected after birth and were therefore affected by intrapartum antibiotic exposure. Prenatal maternal samples might have been more informative but predicting preterm delivery is difficult. The differences in the rates of cesarean section, intrapartum antibiotic exposure and fetal growth restriction between the neonates born by spontaneous or iatrogenic preterm delivery is an obvious concern. Terminating the pregnancy for an iatrogenic cause with a cesarean section delivery is more common in the cases with placental insufficiency and poor intrauterine growth, which could be seen in our patient population. However, potential confounding is ruled out by the fact that the mode of delivery, intrapartum antibiotic exposure or being small for gestational age displayed no association with the preterm gut microbiota in our analyses as independent factors. We may therefore consider our results, where the cause of prematurity emerged as the sole significant factor, reliable.

The preterm neonates in this study exhibited low microbial diversity and great variation in the gut microbiota composition. Uniform colonization patterns could not be seen, which is consistent with previously reported data. High abundance of specific bacteria, including *Enterobacter, Enterococcus* and *Lactobacillus* genus, are reportedly typical for the early preterm gut microbiota.^10^ The most abundant bacterial families in the preterm neonates in our study were Planococcaceae, Lactobacillaceae and Enterobacteriaceae, which all include common pathogenic bacteria. A proinflammatory, low diversity gut microbiota composition has been described earlier as typical for preterm infants.^9^ When compared to the term gut microbiota composition in our study, the preterm gut microbiota composition exhibited significantly lower alpha diversity, significant beta diversity clustering, and higher levels of specific proinflammatory bacteria. As previously reported, preterm infants may have delayed colonization with Bifidobacteria,^23^ which are thought to reduce inflammation and promote a healthy gut composition.^24^ Our findings regarding the microbiota differences in these two groups are therefore consistent with the previously reported data.

The connection between initial gut microbiota composition and health outcomes remains largely unknown. However, data from a study by Arboleya et al.^15^ suggest that the initial gut microbiota composition in preterm infants is associated with growth in the neonatal period. This finding may be of clinical significance since poor postnatal growth, a highly common problem in preterm neonates, has been linked to neurodevelopmental problems later in life. Ensuring adequate growth is a top priority in treating these fragile patients. There are studies suggesting that the early gut microbiota composition of the preterm infant may affect the later gut microbiota composition, which in turn has been linked to several clinical outcomes, including obesity^25^ and allergic diseases.^26^ Our results provide intriguing new information about the establishment and exposures connected to the initial gut microbiota establishment in preterm neonates. Future research should focus on the connections between spontaneous preterm birth, early gut microbiota composition, and short- and long-term health outcomes.

## Supplementary information

Supplementary information
