# Supplementary material for: Spontaneous preterm delivery is reflected in both early neonatal and maternal gut microbiota
Source: Pediatr Res. 2021 Aug 4;91(7):1804–11. doi: 10.1038/s41390-021-01663-8 (PMC9270225; doi:10.1038/s41390-021-01663-8)
Supplement: Supplementary file 1 — Supplementary information [file 41390_2021_1663_MOESM1_ESM.pdf]

Supplementary Figure 1

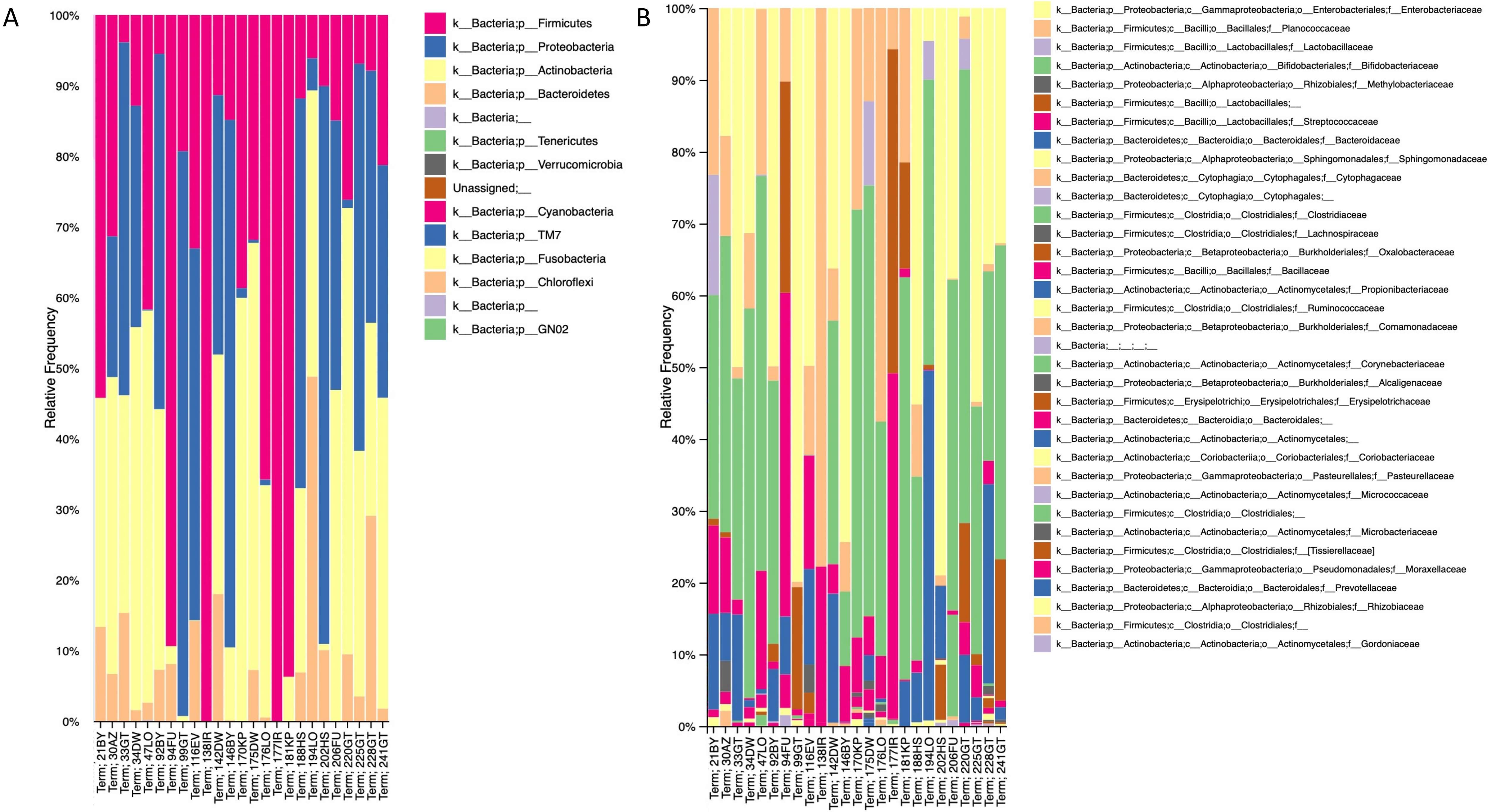

Supplementary Figure 2.

1A. Gestational age	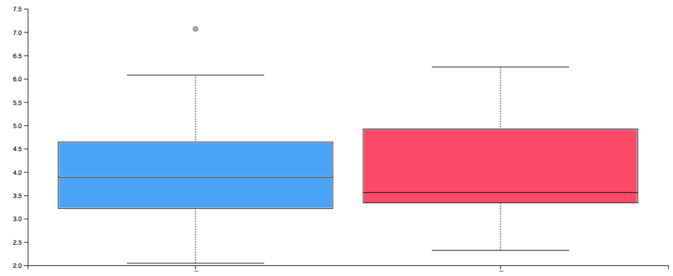	
	GA over 32 weeks (n=38)	GA under 32 weeks (n=17)
	p = 0.74	
1B. Mode of delivery	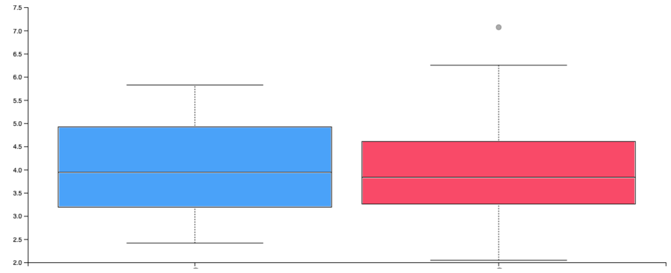	
	sectio (n=21)	vaginal (n=34)
	p = 0.82	
1C. Cause of prematurity	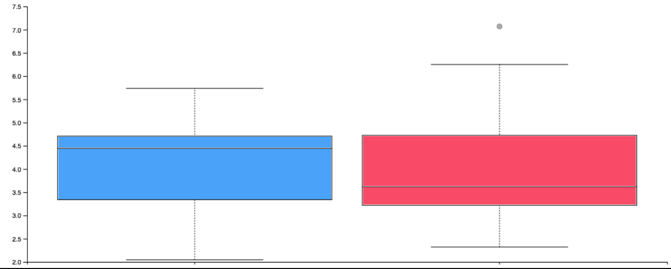	
	iatrogenic (n=21)	spontaneous (n=34)
	p = 0.39	
1D. Maternal antibiotic	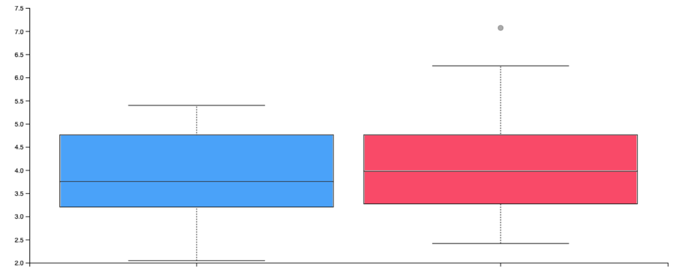	
	no (n=22)	yes (n=32)
	p = 0.66	
1E. Infant antibiotic	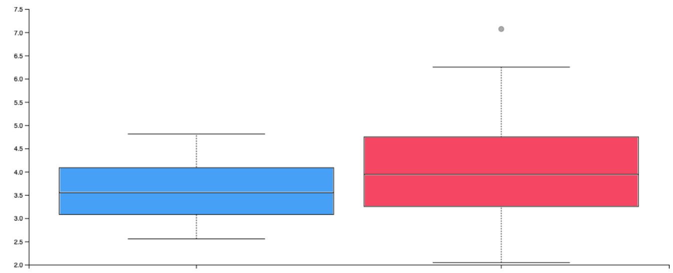	
	no (n=4)	yes (n=51)
	p = 0.54	

Supplementary Figure 3A

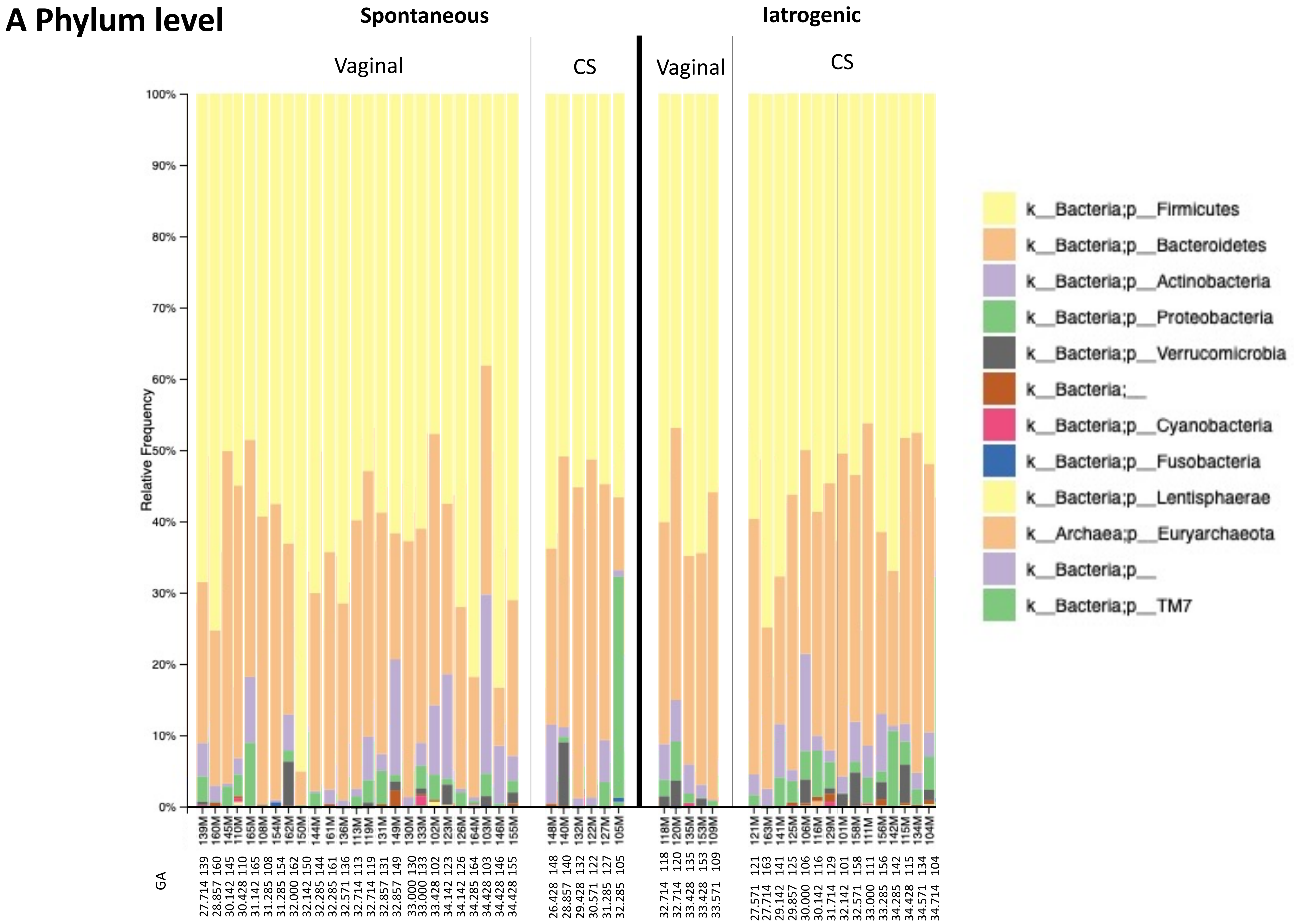

Supplementary Figure 3B

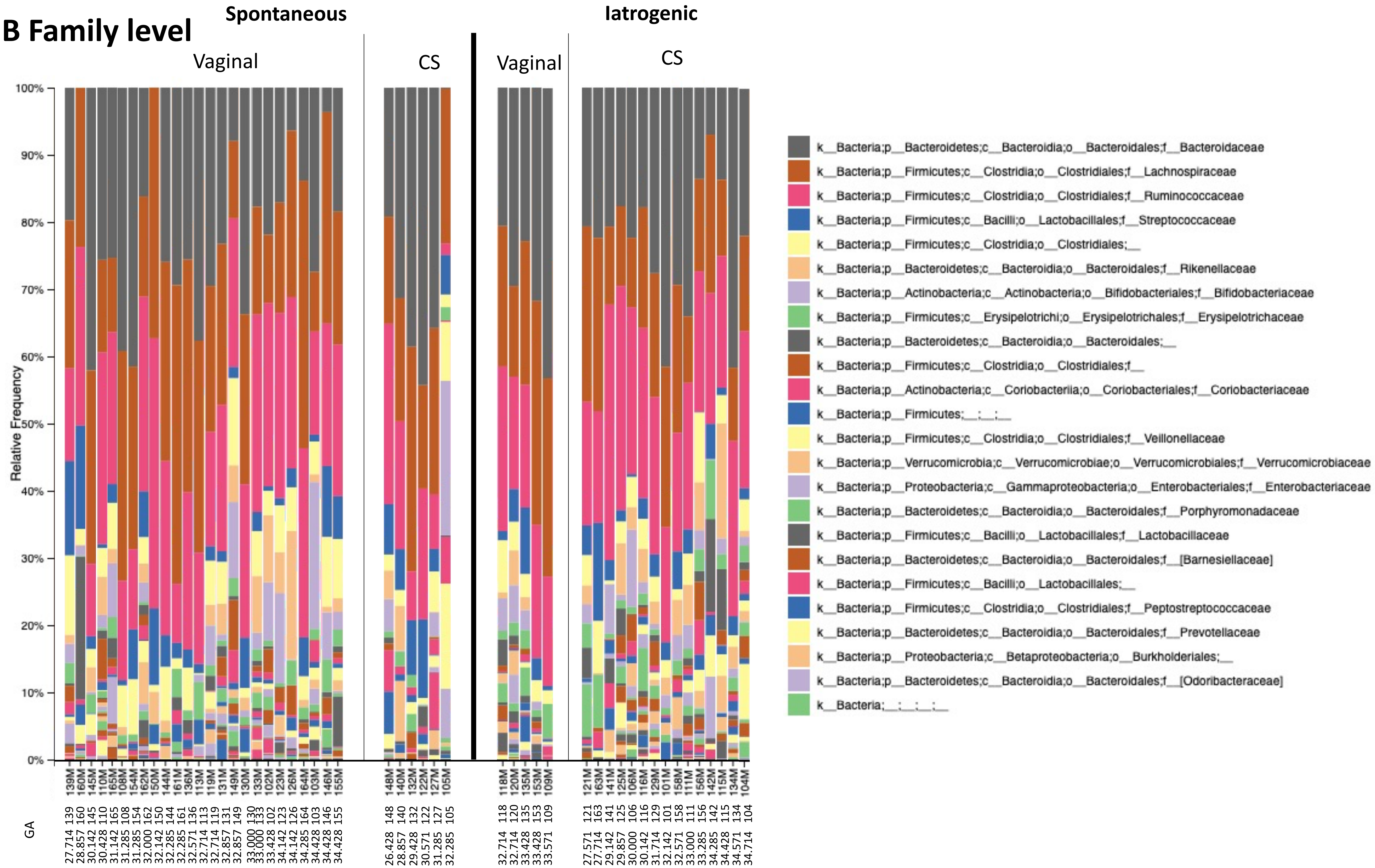

Supplementary Figure 1. Term microbiota taxa plots at (A) phylum and (B) family levels. At the phylum level, the most abundant were Proteobacteria, Firmicutes and Actinobacteria. The three most abundant bacterial families were Bifidobacteriaceae, Enterobacteriaceae and Planococcaceae. The 35 most abundant families are included.

Supplementary Figure 2. Preterm neonate gut microbiota alpha diversity (Shannon). P-values are presented below each figure and calculated with Kruskal-Wallis test. None of the results were statistically significant.

Supplementary Figure 3. Maternal gut microbiota taxa plots in (A) phylum and (B) family levels grouped by gestational age (GA), cause of prematurity and mode of delivery. Firmicutes, Bacteroidetes and Actinobacteria were the most abundant phyla and Bacteroidaceae, Lachnospiraceae and Ruminococcaceae the most abundant bacterial families. The 24 most abundant families are included.
